# Correlation Between Thyroid-Stimulating Hormone (TSH) and Liver Function Test Values in North Karnataka Patients Admitted to a Tertiary Care Hospital

**DOI:** 10.7759/cureus.59004

**Published:** 2024-04-25

**Authors:** Sanjana Murali Krishna, Shreelaxmi V Hegde, Monisha Chellathurai, Nayana Juhy Anitha Mohandas, Swetha Guruswamy, Snehal Pandit, Aysha Afra, Shubra Shetty, Chandu Siripuram

**Affiliations:** 1 Department of Biochemistry, Srinivas Institute of Medical Sciences and Research Centre, Mangalore, IND; 2 Department of Hospital Medicine, Geisinger Community Medical Center, Scranton, USA

**Keywords:** tsh, north karnataka, metabolic regulation, hypothyroidism, comorbidity, blood pressure, liver function test

## Abstract

Background: Thyroid hormones play a pivotal role in regulating metabolic processes, including liver metabolism. The interplay between thyroid function and liver enzymes is complex, with thyroid dysfunction potentially impacting liver function. The relationship between thyroid-stimulating hormone (TSH) levels and liver function parameters is particularly noteworthy, especially in areas like North Karnataka, India, where dietary and environmental factors may impact thyroid disorders.

Aim and objectives: The principal objective of this research is to explore the association between TSH levels and liver function parameters in individuals from North Karnataka. Secondary objectives include examining the relationship between TSH levels, blood pressure, and the prevalence of comorbidities in the study population.

Materials and methods: This retrospective observational study included 75 patients admitted to a tertiary care hospital in North Karnataka. Patients who had undergone both thyroid function tests and liver function tests were included in the study. Data on blood pressure and comorbidities (like hypothyroidism, hyperthyroidism, hypertension, gastritis, alcohol-related liver disease, anemia, chronic obstructive pulmonary disease (COPD), arthritis, diabetes mellitus, migraine, and uterine disorder) were also collected and analyzed.

Results: The study population comprised 48 females (64%) and 27 males (36%), with a mean age of 46.52 years. Thyroid irregularities were observed in 16 patients (21.4%), with hypothyroidism being the predominant thyroid disorder, accounting for 14 cases (18.7%). The distribution of comorbidities included diabetes mellitus (14 patients; 18.7%), anemia (19 patients; 25.3%), migraine (18 patients; 24%), hypertension (17 patients; 22.7%), gastritis (10 patients; 13.3%), COPD (nine patients; 12%), alcohol-related disorders (four patients; 5.3%), arthritis (three patients; 4%), and uterine disorders (eight patients; 10.6%). It is important to note that some patients presented with more than one comorbidity, which may result in an overlap in the total count of specific conditions reported.

Discussion: The absence of a significant correlation between TSH levels and liver function tests in this study contrasts with some previous research, suggesting that regional factors and dietary habits may play a role in these associations. The high prevalence of thyroid disorders, particularly hypothyroidism, underscores the importance of monitoring thyroid function in this population. The presence of comorbidities such as diabetes mellitus and anemia further complicates the clinical picture and highlights the need for comprehensive healthcare approaches.

Conclusion: This study did not find a significant correlation between TSH levels and liver function parameters in patients from North Karnataka. The findings emphasize the need for continued research into the complex interactions between thyroid function and liver metabolism, particularly in regions with unique environmental and dietary influences. Public health initiatives should focus on addressing the high prevalence of thyroid disorders and related comorbidities in this population.

## Introduction

Thyroid hormones are important for controlling metabolism, body weight, energy balance, liver function, and insulin sensitivity. The thyroid gland produces three hormones: calcitonin, triiodothyronine (T3), and thyroxine (T4). Iodine is essential for the synthesis of T3 and T4. The thyroid-stimulating hormone (TSH), produced by the pituitary gland in the brain, regulates the production of these hormones [1, 2].

The liver synthesizes several key proteins that play essential roles in binding and transporting thyroid hormones, such as T4 [1]. The primary protein involved is thyroxine-binding globulin (TBG), which is the major carrier of T4 and T3 in the bloodstream. TBG binds to these hormones with high affinity, thereby regulating their bioavailability and distribution to various tissues. Another important protein is transthyretin (TTR), formerly known as prealbumin, which also binds to T4 but with a lower affinity than TBG. TTR assists in the transport of both thyroid hormones and retinol (Vitamin A) [1, 2]. Additionally, albumin, although it has the lowest affinity for T4 among these proteins, still transports a significant portion of circulating T4 due to its high concentration in the blood [1-3]. Together, these proteins are critical for ensuring that thyroid hormones are adequately available throughout the body to perform their physiological functions. Most thyroid hormones in the blood are attached to proteins. There is a close relationship between the thyroid gland and liver in both health and disease [1]. The liver is important for the activation, transport, and metabolism of thyroid hormones. At the same time, thyroid hormones affect how the liver works. People with underactive (hypothyroidism) or overactive (hyperthyroidism) thyroids can have abnormal liver enzyme levels. Studies by Ajala et al. (2013) [2], Codruta et al. (2015) [3], Sangamesh et al. (2020) [4], Yadav et al. (2013) [5], and Jaafer et al. (2023) [6] have shown connections between liver enzymes (alanine aminotransferase (ALT), aspartate aminotransferase (AST), and alkaline phosphatase (ALP)) and thyroid function.

Age, sex, location, and the amount of iodine in the diet are all factors that affect the prevalence of thyroid disorders in India [7]. Eating iodized salt can help prevent iodine deficiency. However, in some areas of Karnataka (southern state in India), not many people use enough iodized salt. Research has shown that in districts like Bellary, Bijapur, Gulbarga, Kolar, and Shimoga, the use of iodized salt is low [8]. The study participants were patients from North Karnataka who received treatment in a hospital in Mangalore. The main goal of our study was to see if there is a connection between TSH levels and liver function tests (ALT, AST, ALP, total bilirubin (TB), direct bilirubin (DB), indirect bilirubin (IB), total protein (TP), albumin, and globulin). We also looked at the relationship between blood pressure and thyroid and liver functions, as well as other health conditions the patients might have.

## Materials and methods

This retrospective observational study was conducted at a major tertiary care center, focusing on patients aged 18 years and older who received treatment at the facility. The study received approval from the institutional ethics committee (SIMS & RC/ 02/ 22-23). The participants in this study were residents of Vijayanagara, Haveri, and Dhawangere in North Karnataka, who were admitted to the hospital as part of a free treatment camp. The research took place in March 2023 and served as an integral component of the clinical training for third-year medical students, aligning with the newly implemented competency-based medical education (CBME) curriculum.

Data for the study were extracted from the hospital's laboratory records. We included in our analysis only those patients for whom complete data sets were available regarding their thyroid function and liver enzyme levels. Patients with incomplete records were excluded from the study. The data collected encompassed a range of demographic details and clinical parameters, including blood pressure, thyroid function tests (T3, T4, TSH), liver enzyme levels (AST, ALT, and ALP), random blood glucose (RBS) levels, urea, creatinine, and any existing comorbidities. Additional information regarding blood pressure and comorbidities was sourced from the hospital's medical record department.

Statistical analysis

The statistical analysis for this study was conducted utilizing Statistical Package for the Social Sciences (SPSS) software, version 23.0. The relationship between various variables was assessed using the correlation coefficient. The prevalence of comorbidities was determined through descriptive statistics. To compare the differences between groups, the Mann-Whitney U test was employed. A p-value of less than 0.05 was deemed to indicate statistical significance.

## Results

In this research conducted in North Karnataka, India, a total of 75 patients admitted to a tertiary care hospital were analyzed to explore the relationship between TSH levels and liver function parameters. The demographic breakdown of the study population showed a predominance of females, 48 participants (64%), with an average age of 46.52 years (Table 1).

**Table 1:** Demographic breakdown by gender and average age of the participants SD: Standard deviation

Variables	N (%)
Females	48 (64.0%)
Males	27 (36.0%)
Age (years, mean ± SD)	46.52 ± 16.95

The mean TSH level was found to be 3.65 with a SD of 5.45, indicating a wide variance in the data, which is also reflected in the broad SD range from 0.27 to 40.75. This suggests that while the average TSH level is within the typical reference range, there are individuals with levels that deviate significantly from the mean. The T3 levels had a mean of 1.29 and an SD of 0.78, with an SD range from 0.68 to 5.07. Compared to TSH, the T3 levels have a narrower SD range, indicating less variability among the participants' values. The SD range, from 33.05 to 207.25, indicates moderate variability among the subjects. The mean T4 level was 97.12, with an SD of 37.88. Overall, these findings show a diversity in thyroid function among the subjects, with a particularly notable spread in the TSH and T4 values. This may indicate varying degrees of thyroid function or dysfunction within the population studied. The broad ranges for TSH and T4 may also reflect a sample that includes both euthyroid individuals and those with thyroid disease (Table 2).

**Table 2:** Overview of thyroid function test results: statistical data for TSH, T3, and T4 TSH: Thyroid-stimulating hormone, T3: triiodothyronine, T4: tetraiodothyronine

Thyroid variables	Mean + SD	SD range
TSH	3.65 ± 5.45	0.27 - 40.75
T3	1.29 ± 0.78	0.68 - 5.07
T4	97.12 ± 37.88	33.05 - 207.25

**Figure 1:**
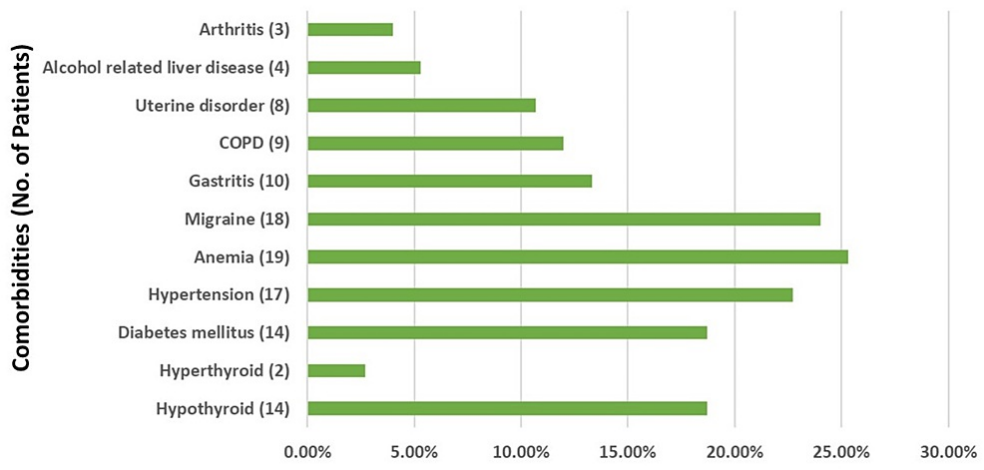
Distribution of comorbidities: hypothyroid, hyperthyroid, hypertension, gastritis, alcohol-related liver disease, anemia, COPD, arthritis, diabetes mellitus, migraine, and uterine disorder COPD: Chronic obstructive pulmonary disease

Comorbidities such as hypothyroidism, hyperthyroidism, hypertension, gastritis, alcohol-related liver disease, anemia, chronic obstructive pulmonary disease (COPD), arthritis, diabetes mellitus, migraine, and uterine disorders were included in the study. Thyroid function tests indicated that 16 (21.4%) of the participants had thyroid abnormalities, with hypothyroidism being the most prevalent condition, affecting 14 (18.7%) of the patients. The distribution of comorbidities in the patient population was diverse. Diabetes mellitus and anemia were the most common comorbidities, present in 14 (18.7%) and 19 (25.3%) of the patients, respectively. Other notable comorbidities included migraine in 18 patients (24%), hypertension in 17 patients (22.7%), gastritis in 10 patients (13.3%), and chronic obstructive pulmonary disease (COPD) in nine patients (12%). Less common conditions were alcohol-related liver disease in four patients (5.3%) and arthritis in three patients (4%) (Figure 1). As noted, some patients exhibited multiple comorbidities, which is reflected in the cumulative total numbers represented in Figure 1.

**Figure 2:**
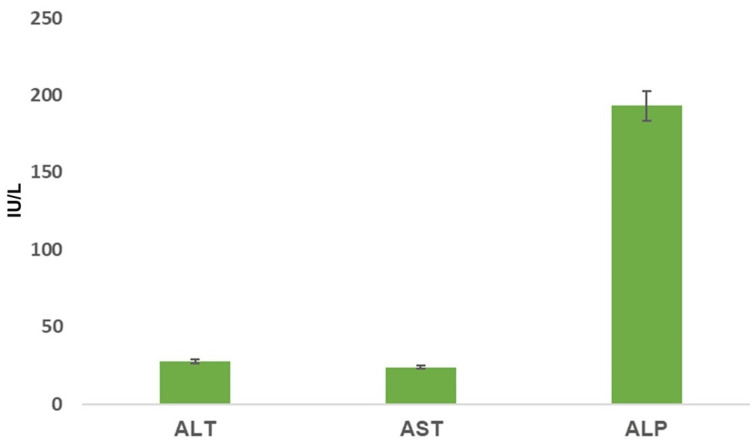
ALT, AST, and ALP levels in the study population ALT: Alanine aminotransferase, AST: aspartate aminotransferase, ALP: alkaline phosphatase, U/L: international units per liter

The levels of liver enzymes like ALT and AST were relatively similar and lower compared to ALP, which was markedly higher. Specifically, the ALT and AST levels were well below 50 units per liter, suggesting that they fall within a normal range. In contrast, ALP levels were significantly higher, averaging just over 200 units per liter, with a notable standard deviation. This disparity may point toward a prevalence of conditions affecting bile flow or bone diseases in the population, as ALP is elevated in these scenarios (Figure 2).

**Figure 3:**
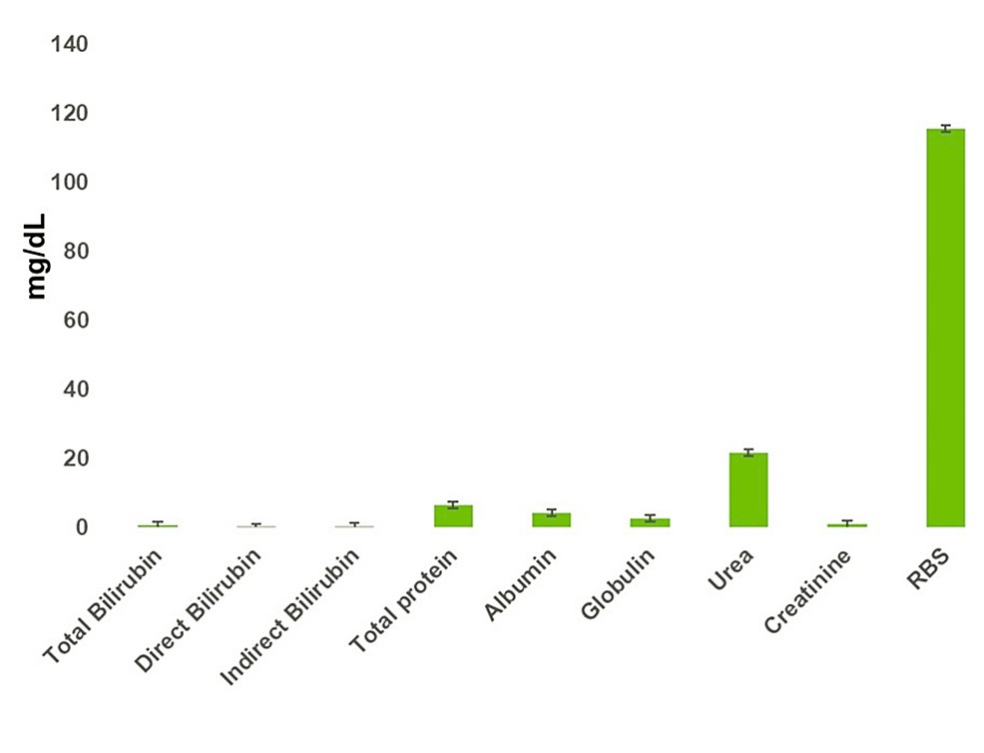
Biochemical variables like total bilirubin, direct bilirubin, indirect bilirubin, total protein, albumin, globulin, urea, creatinine, and RBS in blood sample RBS: Random blood sugar

The biochemical variables like total bilirubin, direct bilirubin, indirect bilirubin, total protein, albumin, and globulin are within relatively narrow ranges, suggesting the liver function is under control. Notably, the levels of urea and creatinine, which are indicators of the kidney function, are modestly elevated, yet still within a typical range. In stark contrast, the level of random blood sugar (RBS) is significantly higher, far surpassing other biochemical values, indicating variability among individuals. This could suggest a trend of glucose regulation issues within the sample, potentially pointing to a prevalence of diabetes or prediabetes conditions among the subjects (Figure 3).

The core analysis of the study focused on the correlation between TSH levels and liver function parameters. The results indicated no significant correlation between TSH and any liver function test parameters, including total bilirubin, direct bilirubin, indirect bilirubin, total protein, albumin, globulin, ALT, AST, and ALP (Table 3). 

**Table 3:** Correlation of TSH with liver function test TB: Total bilirubin, DB: direct bilirubin, IB: indirect bilirubin, TP: total protein, ALB: albumin, GLB: globulin, ALT: alanine aminotransferase, AST: aspartate aminotransferase, ALP: alkaline phosphatase r values calculated using Pearson's correlation coefficient test

Measurement		TB	DB	IB	TP	ALB	GLB	ALT	AST	ALP
TSH	r	0.07	0.09	0.05	-0.19	-0.06	-0.13	0.05	0.02	-0.07

Additionally, the study explored the relationship between blood pressure, TSH levels, and liver function tests and found no significant correlation between these variables (Table 4).

**Table 4:** Correlation of blood pressure with thyroid profile and liver function test Sys BP: systolic blood pressure, Dia BP: diastolic blood pressure, TSH: thyroid-stimulating hormone; T3: triiodothyronine; T4: tetraiodothyronine, TB: total bilirubin, DB: direct bilirubin, IB: indirect bilirubin, TP: total protein, ALB: albumin, GLB: globulin, ALT: alanine aminotransferase, AST: aspartate aminotransferase, ALP: alkaline phosphatase r values calculated using Pearson's correlation coefficient test

Variable		TSH	T3	T4	TB	DB	TP	ALB	GLB	ALT	AST	ALP
Sys BP	r	0.17	-0.18	0.10	0.09	0.13	-0.18	-0.18	-0.14	0.03	0.07	0.01
Dia BP	r	0.07	0.01	0.17	0.05	0.04	0.06	-0.23	0.04	-0.02	0.05	0.17

Further investigation was conducted to compare TSH levels in patients with and without specific comorbidities. The analysis showed that patients with diabetes mellitus had higher mean TSH levels (6.74) compared to those without diabetes (2.88), although the difference was not statistically significant (p = 0.09 (Table 5).

**Table 5:** Comparison of TSH levels when a comorbidity is present or absent COPD: chronic obstructive pulmonary disease, TSH: thyroid-stimulating hormone The Z-test compares the means of two independent groups and calculates the Z-score p-value: The p-value is calculated based on the Z-score; typically, a p-value less than 0.05 is considered significant

Comorbidity	TSH (Present)	TSH (Absent)	Z	p
Diabetes mellitus	6.74 ± 10.72 (14)	2.88 ± 2.73 (57)	1.68	0.09
Hypertension	3.73 ± 4.03 (17)	3.61 ± 5.86 (54)	0.95	0.34
Anemia	2.43 ± 1.87 (18)	4.05 ± 6.17 (53)	1.12	0.26
Migraine	2.47 ± 1.66 (18)	4.04 ± 6.20 (53)	0.70	0.49
Gastritis	2.65 ± 1.64 (10)	3.81 ± 5.84 (61)	0.02	0.99
COPD	7.85 ± 13.53 (9)	3.03 ± 2.66 (62)	0.24	0.81
Uterine disorder	2.63 ± 2.66 (8)	3.77 ± 5.71 (63)	0.93	0.35
Alcohol-related liver disease	2.87 ± 2.84 (4)	3.69 ± 5.58 (67)	0.44	0.67

Similar patterns were observed for other comorbidities, such as hypertension, anemia, migraine, gastritis, COPD, uterine disorders, and alcohol-related liver disease, where no statistically significant differences in TSH levels were found between patients with and without these conditions.

In summary, the study did not demonstrate a significant correlation between TSH levels and liver function parameters or blood pressure in the patient population from North Karnataka. Additionally, the presence of various comorbidities did not significantly affect TSH levels. These findings suggest that in this specific regional context, thyroid function, as measured by TSH levels, may not be directly linked to liver function parameters or influenced by the presence of common comorbidities. Further research with larger sample sizes and more diverse populations may be needed to fully understand the potential relationships between thyroid function, liver function, and comorbidities.

## Discussion

The current study conducted in North Karnataka, India, did not demonstrate a significant correlation between TSH levels and liver function parameters, nor between blood pressure, TSH, and liver function tests. The distribution of comorbidities revealed a high prevalence of hypothyroidism, diabetes mellitus, anemia, and migraine among the patients. Although thyroid dysfunction is commonly associated with diabetes in various studies, the current study did not find significant results in this regard. Additionally, patients with COPD showed higher TSH values, but this was not statistically significant, possibly due to the small sample size.

It was observed that hypothyroidism and diabetes mellitus were the most prevalent endocrine diseases among patients after hypertension. The frequency of anemia and migraine was also very common in the patient population. Thyroid dysfunction is commonly reported in patients with diabetes, and it has been documented in several studies [9-11]. Several studies have shown that serum TSH levels, even within the normal range, are positively associated with hyperglycemia and insulin resistance in euthyroid subjects. This relationship can be explained through various physiological interactions. Firstly, TSH regulates the production of thyroid hormones, which are crucial for metabolic processes, including glucose metabolism and insulin sensitivity [9-12]. Even subtle increases in TSH may indicate a less efficient metabolic state, predisposing individuals to insulin resistance. Higher TSH levels in the normal range are also often linked to higher body mass index (BMI) and adiposity, which can make insulin resistance worse by causing adipose tissue to release adipokines and inflammatory cytokines. TSH levels can also have an impact on the liver, which is crucial for controlling glucose and fat metabolism; elevated TSH may change hepatic enzyme activities, promoting hyperglycemia and insulin resistance [7-12]. Furthermore, TSH may directly impact pancreatic β-cells, influencing insulin secretion and contributing to metabolic imbalances. Genetic factors might also play a role, with variations in genes related to thyroid function or insulin signaling pathways predisposing individuals to these conditions when TSH levels are at the higher end of the normal range [9-11]. This complex interplay suggests that even normal but higher TSH levels could be a risk factor for metabolic syndrome and its related conditions, highlighting the potential need for closer monitoring of TSH levels to manage or prevent these issues effectively. The current study also revealed similar results, but they were not significant. Patients with COPD also presented with higher TSH values, which were not statistically significant. The study's small sample size explains why this is the case.

Other comorbidities prevalent in the patients were gastritis, uterine disorders, alcohol-related liver disease, arthritis, and hyperthyroidism. Hyperthyroidism had a very low prevalence of 2.7, which is comparable with previous studies. The prevalence of hypothyroidism in the present study is in accordance with previous studies [7, 9, 12]. In 100 North Karnataka patients admitted to a tertiary care hospital, Natasha et al. (2023) [12] conducted a similar study using a prospective study design. The prevalence reported by Natasha et al. (2023) was 12% hypothyroidism and 2% hyperthyroidism in patients. The high number of hypothyroid patients seen in hospital admissions today may be due to cultural beliefs, inadequate consumption of micronutrients like iodine, and the consumption of goitrogen foods like cabbage and cauliflower [7].

Literature shows a positive association between thyroid hormones and liver function parameters [2-5]. A recent study by Jaafer et al. (2023) [6] did not show any correlation between TSH, liver enzymes, or serum protein levels. The results of our study conform to the results of Jaafer et al. (2023) [6]. Hypothyroidism and hyperthyroidism significantly affect thyroid function tests. In hypothyroidism, where the thyroid gland is underactive, thyroid hormone levels (T3 and T4) are low, leading to an increase in TSH due to the pituitary gland's attempt to stimulate thyroid hormone production. This condition can also reduce the activity of enzymes like uridine 5'-diphospho-glucuronosyltransferase (UDP) glucuronyl transferase, impacting metabolism. Conversely, hyperthyroidism results from an overactive thyroid gland, causing high levels of thyroid hormones and low levels of TSH. Hyperthyroidism can also increase levels of TBG, affecting hormone transport. Additionally, conditions like cirrhosis can decrease TBG levels due to impaired liver function, complicating the interpretation of thyroid function tests [8-14]. These dynamics illustrate the interconnected nature of thyroid function, enzyme activity, and systemic health.

Recent studies have further explored the relationship between thyroid function and liver health. The First Affiliated Hospital of Xiamen University performed a cross-sectional study from 2017 to 2020 [13], but they could not find a clear link between T3 levels and the severity of liver fibrosis in people with type 2 diabetes mellitus (T2DM) and nonalcoholic fatty liver disease (NAFLD) [13, 14]. Recent research has unveiled that diminished thyroid function is linked to a heightened risk of advanced fibrosis in individuals with metabolic dysfunction-associated fatty liver disease (MAFLD), indicating that thyroid activity may play a role in liver health​ [15-18]​. Furthermore, studies on morbidly obese patients showed a positive association between TSH and the risk of hepatic steatosis, as well as between higher levels of free thyroxine (FT4) and lower levels of free triiodothyronine (FT3) with higher levels of total bilirubin​ [19-23].

These findings highlight the complex interplay between thyroid function and liver health, which may vary depending on the population studied and the specific liver conditions considered. The current study's lack of significant findings could be attributed to its retrospective nature, small sample size, and single-center design. Future larger population-based studies are necessary to establish the epidemiology of thyroid disorders and their clinical implications in hospital settings, considering the potential influence of thyroid function on liver health as suggested by recent research. 

Also, the outcome of our study is from a single-center study design. This was an undergraduate student research with barriers to students like inadequate time, priorities like university education, and lack of funding. With the available resources in hand, the study was conducted. Future larger population-based studies are required to establish the epidemiology of thyroid disorders and their clinical implications in hospital settings.

Limitations

Firstly, being a retrospective study, it relies on previously recorded data, which might not have been collected with the specific aim of this research, potentially leading to incomplete or biased data. Secondly, the small sample size of only 75 patients limits the statistical power of the study and may not provide a representative view of the general population. Thirdly, as a single-center study, the findings might not be generalizable to other regions or healthcare settings due to differences in patient demographics, healthcare practices, and environmental factors. Additionally, the cross-sectional nature of the study does not allow for the assessment of temporal relationships between thyroid function, liver parameters, and blood pressure, which would require a longitudinal study design to establish causality. The study might also not have adequately controlled for all potential confounding factors, such as medication use, alcohol consumption, and other lifestyle factors, that could influence the relationship between TSH levels and liver function parameters. Moreover, the study focused primarily on TSH levels and did not extensively explore other aspects of thyroid function, such as free T4 and T3 levels, which could provide a more comprehensive understanding of thyroid health and its association with liver function. Lastly, variability in liver function test interpretation, which can be influenced by various factors and differ between laboratories, could affect the consistency of the results. Future research with larger, multi-center, prospective studies that control for potential confounders and explore a broader range of thyroid function parameters is needed to validate and expand upon these findings.

## Conclusions

The research conducted on patients at a tertiary care hospital in North Karnataka found no significant link between TSH levels and liver function tests, or between TSH levels and blood pressure. The study did, however, uncover a high prevalence of endocrine issues, notably thyroid disorders and diabetes mellitus. There was also a notable occurrence of anemia, migraine, and hypertension among the participants. These findings stress the need to consider regional dietary habits and environmental factors when assessing the influence of thyroid function on liver metabolism. The study calls for expanded multicentric research involving larger cohorts to verify these findings and to deepen our understanding of the connections between thyroid function, liver health, and comorbid conditions in this demographic.
